# The role of domain-general cognitive control in language comprehension

**DOI:** 10.3389/fpsyg.2014.00335

**Published:** 2014-04-28

**Authors:** Evelina Fedorenko

**Affiliations:** Psychiatry Department, Massachusetts General HospitalCharlestown, MA, USA

**Keywords:** multiple-demand system, cognitive control, fMRI, sentence processing, language, modularity

## Abstract

What role does domain-general cognitive control play in understanding linguistic input? Although much evidence has suggested that domain-general cognitive control and working memory resources are sometimes recruited during language comprehension, many aspects of this relationship remain elusive. For example, how frequently do cognitive control mechanisms get engaged when we understand language? And is this engagement necessary for successful comprehension? I here (a) review recent brain imaging evidence for the neural separability of the brain regions that support high-level linguistic processing vs. those that support domain-general cognitive control abilities; (b) define the space of possibilities for the relationship between these sets of brain regions; and (c) review the available evidence that constrains these possibilities to some extent. I argue that we should stop asking *whether* domain-general cognitive control mechanisms play a role in language comprehension, and instead focus on characterizing the division of labor between the cognitive control brain regions and the more functionally specialized language regions.

Language is one of few cognitive abilities unique to our species. However, language has neither evolved nor does it exist in isolation from other cognitive and neural machinery (e.g., Christiansen and Chater, 2008; cf. Fodor, 1983), which may be largely shared between humans and non-human animals (e.g., van Horik and Emery, 2011; Kaas, 2013). This machinery includes sensory and motor systems, memory and attention mechanisms, and mechanisms that support social cognition, among others. This paper examines the relationship between high-level language processing and domain-general cognitive control, with a focus on the *brain systems* that support these cognitive capacities.

Brain regions that support domain-general cognitive control have been implicated in a wide range of goal-directed behaviors (see e.g., Duncan, 2010, for a recent review). In the domain of language, cognitive control has been shown to play an important role in language *production*, based on behavioral evidence (e.g., Alm and Nilsson, 2001; Roelofs and Piai, 2011; Strijkers et al., 2011), brain imaging studies (e.g., Müller et al., 1997; Ojemann et al., 1998; Indefrey and Levelt, 2004; Kerns et al., 2004; Haller et al., 2005; Shuster and Lemieux, 2005; Alario et al., 2006; Bohland and Guenther, 2006; Shapiro et al., 2006; Basho et al., 2007; Harrington et al., 2007; Troiani et al., 2008; den Ouden et al., 2009; Eickhoff et al., 2009; Wilson et al., 2009; Brendel et al., 2010; Tremblay and Small, 2011; Adank, 2012; Geranmayeh et al., 2012; Grande et al., 2012; Heim et al., 2012; Delnooz et al., 2013) and investigations of patients with brain damage (e.g., Ziegler et al., 1997; Nestor et al., 2003; Ash et al., 2010; Wilson et al., 2010; Baldo et al., 2011; Coelho et al., 2012; Endo et al., 2013). Indeed, planning and producing linguistic utterances bears intuitive similarity to non-linguistic goal-directed behaviors like reaching (e.g., Bernstein, 1996; Culham and Valyear, 2006; Grafton and Hamilton, 2007; Ridderinkhof et al., 2011) or playing a musical instrument (e.g., Meister et al., 2004). In contrast, language *comprehension* (i.e., the process of extracting meaning from the linguistic signal) is, or at least can be, a more “passive,” automatic process: just like we can't help but recognize a face upon seeing a face-like configuration (e.g., Suzuki and Cavanagh, 1995; see Palermo and Rhodes, 2007 for a review), we often can't help but interpret linguistic input if we know the language in question (e.g., Fodor, 1983; Pinker, 1994; Shtyrov and Pulvermüller, 2007; Pulvermüller et al., 2008; Wild et al., 2012). That said, much behavioral and neuroimaging evidence (to be reviewed in section Narrowing Down the Hypothesis Space for the Relationship between Language Processing Mechanisms and Cognitive Control Mechanisms) suggests that domain-general cognitive control mechanisms do sometimes get recruited during language *comprehension*. In this position paper, I discuss two inter-related aspects of the relationship between language processing and cognitive control that are not yet well-understood: (i) when (i.e., under what circumstances) the cognitive control mechanisms get engaged during language understanding; and (ii) whether this engagement is necessary for comprehension (i.e., whether understanding linguistic input *requires* domain-general cognitive control mechanisms, or whether those mechanisms are helpful but non-essential).

The paper is structured as follows: First, I introduce the brain regions that support high-level language processing vs. domain-general cognitive control, and discuss the evidence for the neural separability of these two sets of brain regions. I then introduce two questions about the relationship between language comprehension and cognitive control and define the hypothesis space for each. I then proceed to discuss the arguments and evidence—from behavioral and brain imaging work in healthy and brain-damaged populations—that constrain these hypotheses. Finally, I summarize and conclude.

## High-level language processing brain regions and domain-general cognitive control brain regions

### High-level language-processing brain regions

**Figure 1:**
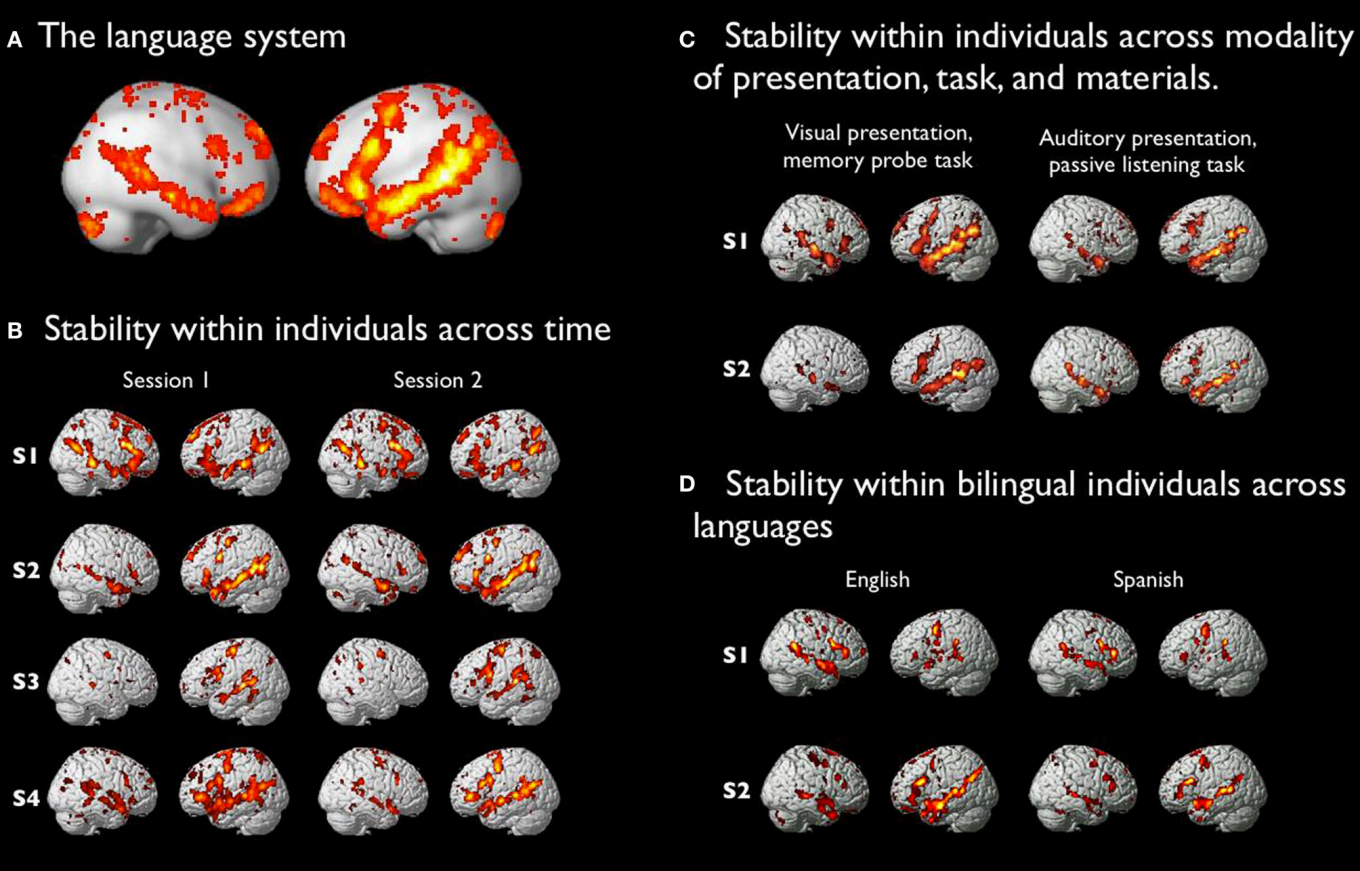
**(A)** The language system: a set of brain regions that are robustly and consistently activated by linguistic input (see (Fedorenko and Thompson-Schill, 2014); Fedorenko and Thompson-Schill, for further discussion of how to define the “language system/network”). A probabilistic activation overlap map for the contrast between sentences and sequences of pseudowords (adapted from Fedorenko et al., 2010). Warmer colors indicate greater proportions of subjects showing a reliable sentences > pseudoword lists effect. **(B)** Activation maps for four sample subjects tested on the sentences > pseudoword lists contrast across two independent scanning sessions, between 1 and 6.5 months apart. (For subjects 2 and 4, non-overlapping sets of materials were used across the two sessions). **(C)** Activation maps for two sample subjects for a contrast between sentences and pseudoword lists presented visually with a memory-probe task (participants had to decide after each sentence or pseudoword sequence whether the probe word/pseudoword appeared in the preceding stimulus), and a contrast between sentences and pseudoword lists (with non-overlapping materials) presented auditorily with a passive listening task. **(D)** Activation maps for two sample English-Spanish bilingual subjects for a contrast between sentences and pseudoword lists in the two languages. (The materials across the two languages were not related to each other in any way, so the similarity is not likely to be due to similar semantic content).

A number of regions in the human brain robustly respond to linguistic input. These regions include most prominently regions on the lateral surface of the left frontal, temporal and parietal cortices, but also a number of other cortical, subcortical and cerebellar regions (Figure 1A). Originally discovered in patients with brain damage (e.g., Broca, 1861; Dax, 1863; Wernicke, 1874/1969; Geschwind, 1970), these regions have been observed in PET and fMRI since the earliest days of brain imaging research (e.g., Petersen et al., 1988; Petersen and Fiez, 1993; Binder et al., 1997). These regions are consistent (albeit variable in their exact topography; e.g., Fedorenko et al., 2010) across individuals (e.g., Frost et al., 1999; Allendorfer et al., 2012), languages (e.g., Chee et al., 1999a,b; Illes et al., 1999; Klein et al., 1999; Hernandez et al., 2001; Pu et al., 2001; Hasegawa et al., 2002; Chee et al., 2003; Mahendra et al., 2003; Briellmann et al., 2004; see e.g., van Heuven and Dijkstra, 2010 and Sebastian et al., 2011 for reviews), modality of presentation (e.g., Chee et al., 1999c; Pinel et al., 2007; Buchweitz et al., 2009; Fedorenko et al., 2010; Braze et al., 2011) and developmental experiences, including complete sensory deprivation in the auditory or visual modality (Neville et al., 1998; Newman et al., 2010; Bedny et al., 2011). Furthermore, these regions can be quickly (in ~10–15 min) and reliably identified in individual participants (Fedorenko et al., 2010), and they are stable within an individual over time (Figure 1B; Fedorenko et al., 2010; Mahowald and Fedorenko, in preparation), as well as being robust to changes in the materials, modality of presentation, and task (Figure 1C), and language for bilingual speakers (Figure 1D).

This set of brain regions can be identified with a variety of contrasts that compare a more language-like stimulus with a less language-like stimulus^1^ (e.g., words vs. fixation or tones—Binder et al., 1997; Diaz and McCarthy, 2009; words vs. pseudowords—Petersen et al., 1990; sentences vs. fixation—Kuperberg et al., 2003; sentences vs. false font or consonant strings—Bavelier et al., 1998; Robertson et al., 2000; Noppeney and Price, 2004; sentences vs. lists of words or pseudowords—Snijders et al., 2009; Fedorenko et al., 2010; Fedorenko and Kanwisher, 2011). Language-like-ness can be operationalized in terms of the amount of overlap between the stimulus and natural language. For example, phonotactically legal pseudowords and words match the sound-level properties of natural language, real words further match the lexical representations, and phrases or sentences match both lexical representations as well as larger structural / meaning units. And the process of language comprehension can be thought of, at least in part, as finding matches between the input and the stored language knowledge representations, with more/better matches leading to greater responses. In the remainder of the paper I will refer to this set of brain regions as the “language system”^2^^,^^3^.

The stability of language activations within individuals across time and their robustness to variation in many properties of the defining contrast suggest that the language system may constitute a “natural kind,” i.e., a meaningful and stable subset of the brain. Two further lines of evidence suggest that these regions constitute an integrated functional system^4^. The first comes from studies of resting-state functional correlations, often referred to as “functional connectivity” (e.g., Fox and Raichle, 2007). In particular, the entire language system discussed above consistently emerges in the analyses of low-frequency oscillations across the brain during rest (e.g., Turken and Dronkers, 2011; Newman et al., 2013; Blank et al., submitted; see e.g., Catani et al., 2005, for DTI data consistent with the idea that these regions form a network). Although the interpretation of resting-state correlation patterns is still debated, these correlations appear to capture stable aspects of the functional organization of the human brain that persists across different mental states including sleep (e.g., Horovitz et al., 2008) and anesthesia (e.g., Vincent et al., 2007), and in some cases goes beyond known anatomical connections (e.g., Honey et al., 2009).

The second line of evidence comes from investigations of cortical thinning patterns in primary progressive aphasia, a neurodegenerative condition that disproportionately, and perhaps selectively, affects language processing (e.g., Mesulam, 2001; Grossman and Ash, 2003; Gorno-Tempini et al., 2004). The pattern of cortical thinning in this disorder—especially in the semantic variant—is strikingly similar to the functional activations for the contrasts, like e.g., *sentences > pseudoword lists* (e.g., Listerud et al., 2009; Rohrer et al., 2009; Dickerson, 2011; Gorno-Tempini et al., 2011; Rogalski et al., 2011). The precise mechanisms of degeneration constitute an area of active research, but one influential proposal that has been put forward argues for propagation along transsynaptic connections (Seeley et al., 2009).

In summary, a set of regions in the human brain (a) robustly respond to language input (with responses decreasing as the stimulus becomes less language-like, or when attention is drawn away from the linguistic properties of the stimulus and toward its perceptual features); (b) show strong correlations in their time courses during rest; and (c) are jointly susceptible to neurodegeneration in primary progressive aphasia. Together, these sets of findings suggest that these regions constitute a functional system. Given that these brain regions get activated by linguistic input and given that damage to these regions in mature brains leads to language deficits (e.g., Damasio, 1992; Bates et al., 2003), it is natural to assume that they play an important (and causal) role in interpreting the linguistic signal, although some components of this system have been argued to not be exclusively engaged by language but to instead support more abstract semantic processing (e.g., Hagoort et al., 2004; Patterson et al., 2007; Binder et al., 2009).

### Domain-general cognitive control brain regions

**Figure 2:**
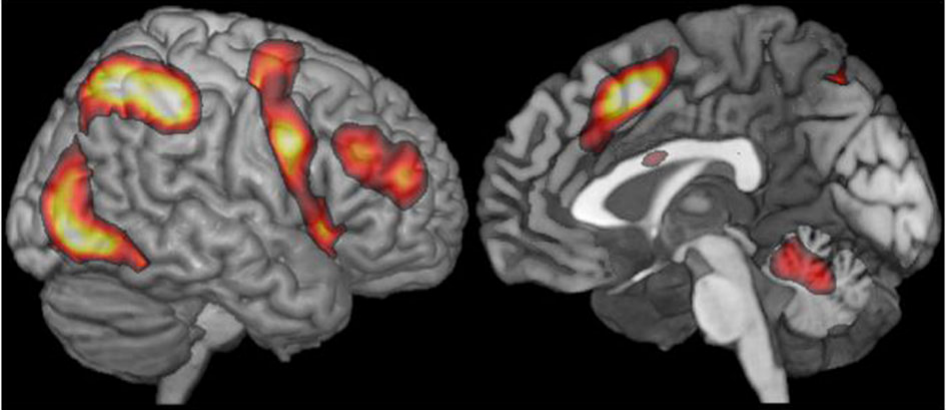
**From Fedorenko et al. (2013).** A group-level representation of the multiple-demand activity based on average activity in left and right hemispheres. Following reflection of left hemisphere data to the right, 14 (7 tasks × 2 hemispheres) *t*-maps were averaged, and the resulting map was thresholded at *t* = 1.5. The tasks included: arithmetic addition, spatial working memory, verbal working memory, multi-source interference task (MSIT; Bush and Shin, 2006), a verbal version of MSIT, and Stroop (data from Fedorenko et al., 2013).

A number of regions in the human brain have been implicated in a broad range of goal-directed behaviors (e.g., Posner and Petersen, 1990; Cabeza and Nyberg, 2000; Corbetta and Shulman, 2002; Cole and Schneider, 2007; Duncan, 2010). These regions include parts of the dorsolateral prefrontal cortex (along the inferior frontal sulcus/middle frontal gyrus), parts of the insular cortex, regions along the precentral gyrus (going inferiorly to the posterior aspects of the inferior frontal gyrus, IFG), pre-supplementary and supplementary motor area, parts of the anterior cingulate, and regions in and around the intraparietal sulcus (Figure 2). This set of regions—with sometimes slightly differing inclusion criteria and/or subdivisions—is referred to in the literature by many names, including “task-positive network,” “cognitive control network,” “fronto-parietal attention network,” and “multiple-demand (MD) system.” Following Duncan (2006, 2010), I will use the term “multiple-demand (MD) system” throughout the paper, but none of the arguments in section Narrowing Down the Hypothesis Space for the Relationship between Language Processing Mechanisms and Cognitive Control Mechanisms hinge on the details of any particular proposal about these regions.

In brain imaging investigations, difficulty contrasts across many manipulations have been shown to activate the MD system. For example, Duncan and Owen (2000; also Duncan, 2006) performed a meta-analysis of activation peaks from neuroimaging studies that manipulated (i) the number of items held in memory (more vs. fewer items), (ii) the duration of holding information in memory (long vs. short), (iii) inhibitory demands (high vs. low), (iv) task novelty (new vs. practiced tasks), and (v) perceptual difficulty (difficult to perceive vs. easy to perceive). Across all of these manipulations, activations were observed in the frontal and parietal MD regions. More recently, Fedorenko et al. (2012, 2013; see also Wojciulik and Kanwisher, 1999; Stiers et al., 2010) provided evidence for overlap among diverse demanding tasks at the single-subject level, ruling out the possibility that the overlapping regions that emerged in the earlier meta-analyses were simply an artifact of spatial averaging across studies (e.g., Nieto-Castañon and Fedorenko, 2012). Furthermore, a set of brain regions that very much resembles the MD system also emerges in the resting-state correlation data (e.g., Power et al., 2011).

Even stronger evidence of domain-generality comes from single-cell recording studies in non-human primates, which have shown that many neurons in the frontal lobes exhibit substantial flexibility, varying their response properties according to task demands (e.g., Freedman et al., 2001; Miller and Cohen, 2001; Duncan, 2001; Cromer et al., 2010). For example, Freedman et al. (2001) trained macaques to categorize visual stimuli according to one dimension (cats vs. dogs). Following training, a substantial proportion of frontal neurons responded categorically to the relevant dimension. However, after training on a new task that used the same stimuli but required attention to a different dimension of the stimuli, the same neurons that previously categorized stimuli into cats and dogs now showed categorical responses to the new task-relevant dimension (see also Roy et al., 2010). These and other results suggest that these frontal neurons adapt the information they code to fit current goals. Similar “adaptive coding” has been reported in the parietal cortex (Freedman and Assad, 2006).

How do MD regions support complex behaviors? As of now, this remains an open question. Some notions that have been prominent in the literature in the context of this system include attention (e.g., Posner and Petersen, 1990; Desimone and Duncan, 1995; Petersen and Posner, 2012), working memory (e.g., Goldman-Rakic, 1995), cognitive control (e.g., Miller and Cohen, 2001; Koechlin et al., 2003; Badre and D'Esposito, 2009), structure building/unification (e.g., Hagoort, 2005), timing and/or sequencing (e.g., Luria, 1966; Janata and Grafton, 2003; Fuster, 2008), attentional episodes in goal-directed behavior (Duncan, 2010), and conscious awareness (e.g., Dehaene and Changeux, 2011), among others. However, most existing proposals are generic enough to be compatible with a wide range of data patterns. Nonetheless, whatever the precise computations conducted by the MD regions turn out to be (see e.g., Rigotti et al., 2010, for a proposal), this system is clearly of fundamental importance to humans, having been causally linked to fluid intelligence (Woolgar et al., 2010).

Given the spatial extent of the MD system and the cytoarchitectonic and connectomic diversity of its regions, many researchers have attempted to divide the MD system into sub-systems (e.g., Botvinick et al., 2001, 2004; Koechlin et al., 2003; Dosenbach et al., 2007; Badre and D'Esposito, 2009), and/or to map specific components of the system onto particular mental functions [e.g., Aron et al. (2004) argued that the right IFG plays a critical role in cognitive control; Novick et al. (2005) made a similar argument for the left IFG]. Claims of dissociations among different executive functions have also been made based on behavioral work in healthy participants (individual differences and dual-task paradigms; e.g., Engle et al., 1999; Miyake et al., 2000) and in brain-damaged individuals (e.g., Vallar and Shallice, 1990; Hamilton and Martin, 2005). Correlations across regions in resting functional data have also been taken to argue for a fractionation of this system (Power et al., 2011). However, the broad similarity in functional responses among the MD brain regions is striking. As a result, for the purposes of this paper I consider the MD system as a whole, while allowing for the possibility that only a subset of this system may end up being important for language (see section The Hypotheses Space for the Relationship Between Language Processing Mechanisms and Cognitive Control Mechanisms).

### Neural separability of high-level language processing brain regions and domain-general cognitive control brain regions

In recent work we investigated the relationship between high-level language processing brain regions and domain-general cognitive control brain regions (Fedorenko et al., 2011): we defined the regions of the language system using the *sentences > pseudoword lists* contrast and then examined the responses of those functionally-defined regions of interest (fROIs) to a number of non-linguistic cognitive tasks that have been previously argued to share machinery with language processing. With the exception of one region (the LMFG fROI), language-responsive regions showed no response to arithmetic processing (see also Monti et al., 2012), working memory, or cognitive control tasks, like Stroop. However, the latter set of tasks robustly activated the MD system, whose subsets are located in close proximity to language-responsive regions, especially in the left frontal lobe (Fedorenko et al., 2012). Based on these results, we argued that language-responsive regions are functionally specialized for linguistic processing and require language input to drive them.

There are at least three possible objections to these results and their interpretation, and I attempt to briefly address them here. The first objection is as follows: perhaps some regions of the MD system do respond to sentences more than pseudoword lists—just like the language regions do—but the response is weaker and/or more variable across individuals. This would lead us to miss some language-responsive regions and thus to potentially miss overlap between responses to language and demanding cognitive tasks. In Fedorenko et al. (2011) we tried to ameliorate this concern by performing a whole-brain search—at liberal thresholds—for overlap between the responses to the language contrast (*sentences > pseudoword lists*) and the *hard > easy* contrast in each of the non-linguistic demanding tasks. This search did not reveal much beyond what the basic analysis of the language-responsive fROIs had already shown: (i) language and verbal working memory showed overlap in the LMFG fROI; and (ii) there was a small region of overlap between language and two of the tasks (verbal working memory and Stroop) in posterior and dorsal-most part of the LIFG fROI, possibly due to the fact that MD regions abut the language-responsive parts of LIFG dorsally and posteriorly (see also Fedorenko et al., 2012), with spatial smoothing leading to the appearance of overlap (this possibility remains to be tested empirically using high-resolution scanning of the frontal cortex; cf. Schwarzlose et al., 2005). In Fedorenko et al. (2013; see also Supplementary Material), we report an analysis that shows that MD regions in fact respond to sentences and pseudowords in the *opposite way* from the language regions: they respond more strongly to linguistically degraded stimuli (pseudoword lists) than to linguistically meaningful and structured stimuli (sentences), suggesting that the language and the MD systems are spatially and functionally distinct.

The second objection is that perhaps the sentences we use in our “language localizer” task (Fedorenko et al., 2010) are too simple and don't contain a sufficient number of features that have been shown to cause comprehension difficulty, such as non-local dependencies (e.g., Gibson, 1998; Grodner and Gibson, 2005), lexical and/or structural ambiguity (e.g., Frazier, 1987; MacDonald et al., 1994), or low-frequency words or constructions (Preston, 1935; Forster and Chambers, 1973; Jurafsky, 1996; Levy, 2008). Maybe if more such features were present in the sentences, we would observe greater overlap between language and MD activations, which would manifest as (a) a greater response to MD tasks in the language regions, and/or (b) a greater response to sentences than pseudoword lists in the MD regions. In Supplementary Material, we show that even when we use naturalistic language materials (from the Brown corpus; Kucera and Francis, 1967) that are representative of the kind of input that our language comprehension system receives, we find similar non-overlap between the activations for the language localizer contrast and demanding cognitive tasks.

Finally, the third objection is as follows: given that (a) we typically use a memory probe task in our language localizer (where after each sentence or sequence of pseudowords participants have to decide whether a probe word/pseudoword appeared in the preceding stimulus; Fedorenko et al., 2010), and (b) the memory probe task is more difficult in the control condition (pseudoword lists) than in the sentences condition, we may be biasing ourselves against finding overlap with demanding tasks because, by design, we are excluding regions that respond to general cognitive effort. To investigate this possibility, we compared responses to a demanding task (spatial working memory) in fROIs defined by two different versions of the language localizer (with vs. without the memory probe task; see Supplementary Material). Across both versions of the localizer, we found little or no response to the conditions of the spatial working memory task in the language fROIs, similar to what we originally reported in Fedorenko et al. (2011). This result is not surprising given the similarity in the topographies of activations for different versions of the language localizer (see Figure 1).

In summary, regions of the language system are spatially and functionally distinct from the domain-general MD system. In contrast to the language regions, the MD regions respond at least as much, or more, during the processing of unconnected meaningless elements (pseudowords) as during the processing of sentences, including naturally occurring ones. The most important *implication* of the spatial segregation between the language system and the MD system is that we need to distinguish between the two in characterizing their roles in language comprehension/production, because the computations they perform are likely to be different given their different response profiles. This is especially important in the left frontal lobe, where subsets of each system reside side-by-side within Broca's area (Fedorenko et al., 2012).

Before proceeding to the next section, two conceptual issues that sometimes get conflated in the literature are important to clarify. First, functionally specialized circuits (e.g., brain regions that selectively respond to linguistic input) need not be *encapsulated* (see e.g., Coltheart, 1999; Barrett and Kurzban, 2006 for discussion). Our brain is highly interconnected, although some brain regions have been argued to be more globally connected than others, serving as “hubs” (e.g., Achard et al., 2006; Sporns et al., 2007; Hagmann et al., 2008; Heuvel et al., 2008; Buckner et al., 2009; Cole et al., 2010). Given this interconnectivity, the notion of encapsulation is a priori not plausible as applied to language-responsive or any other brain regions. Moreover, apart from perhaps quite obvious interactions between high-level language processing regions and sensory (visual and auditory) regions as well as motor regions that support articulation or control eye-movements during reading, abundant evidence shows that the language system interacts with many higher-level cognitive systems, including the visual system (e.g., Myachykov and Posner, 2005; Ferreira and Tanenhaus, 2007), the system that supports social cognition (e.g., Brennan et al., 2010; Fitch et al., 2010), and the domain-general working memory/cognitive control mechanisms (as will be discussed in section Narrowing Down the Hypothesis Space for the Relationship between Language Processing Mechanisms and Cognitive Control Mechanisms). So, although lack of encapsulation of the language system has sometimes been offered as an argument against domain-specificity of language (e.g., Blumstein and Amso, 2013), this argument does not hold because functional specialization is perfectly compatible with interactions between specialized mechanisms and the rest of the mind and brain (see also Fedorenko and Thompson-Schill, 2014).

And second, specialized circuits need not be innate (see e.g., Karmiloff-Smith, 1992; Elman et al., 1996 for discussion). Functional specialization can develop via extensive experience with particular stimuli. One notable example is the visual word-form area, vWFA, a visual region that responds selectively to letters in one's native script (e.g., Baker et al., 2007). Recent work with non-human primates also suggests that specialized circuits can develop via an experiential route (e.g., Srihasam et al., 2011). Given that language is one of the most frequent and salient stimuli in our environment from birth (or even before) and throughout our lifetimes, it is computationally efficient to develop machinery that is specialized for processing linguistic stimuli.

## The hypotheses space for the relationship between language processing mechanisms and cognitive control mechanisms

I focus on two inter-related aspects of the relationship between language processing mechanisms and cognitive control mechanisms.

First, how frequently do cognitive control mechanisms get engaged when we understand language? The logical possibilities here range from never to always. Previous evidence (to be discussed in section Narrowing Down the Hypothesis Space for the Relationship between Language Processing Mechanisms and Cognitive Control Mechanisms) has established that cognitive control mechanisms are sometimes engaged when we understand language, thus ruling out the “never” possibility. However, this still leaves us with a large space of possibilities, from engagement only in rare circumstances, to continual engagement whenever we understand language.

And second, is the engagement of cognitive control mechanisms necessary for understanding language? The at least occasional engagement of domain-general cognitive control mechanisms in language comprehension is compatible with, but does not entail, their *necessity* for comprehension.

My working definition of “necessary” is as follows: A brain region is necessary for a mental process *x*, if and only if *x* cannot proceed (or proceeds with substantially reduced speed or accuracy) once the relevant brain region is damaged or removed^5^. So, a brain region is necessary for language comprehension if and only if linguistic input cannot be interpreted without this region. (Note that the necessary role of a brain region in a mental process is orthogonal to its functional specialization for that mental process. A brain region may be necessary for processing a particular class of stimuli and yet be engaged in processing a wide range of stimuli. For example, primary visual cortex is critical for face perception and yet it is engaged during the processing of any visual stimulus).

At least four possibilities exist with respect to the question of whether cognitive control mechanisms—that I assume to be implemented in the MD system, as discussed above—are critical for understanding language:

1. *Every component of the MD system is necessary for language comprehension*.
  If this were the case, then disrupting any part of the MD system would lead to severe difficulties in understanding language.
2. *Only some components of the MD system (e.g., perhaps only the MD regions in the left hemisphere; Duncan, 2001) are necessary for language comprehension*.
  According to this possibility, disrupting some but not other parts of the MD system would lead to severe comprehension problems.
3. *The MD system as a whole is necessary for language comprehension, but no individual component is critical (i.e., the “responsibilities” are distributed across the system)*.
  This possibility is inspired by the findings of Woolgar et al. (2010), who demonstrated a linear relationship between the amount of damage to the MD system and the intelligence quotient (IQ), such that the more extensive the damage the lower the IQ. A similar relationship may hold between the MD system and language comprehension: disrupting any individual component may only slightly affect comprehension abilities, but disrupting increasingly larger portions of the MD system would eventually lead to one's inability to comprehend linguistic input. This general idea is reminiscent of Lashley's (1929) notion of equipotentiality, which may to some degree characterize the MD system.
4. *No part of the MD system is critical for language comprehension*.
  If this were the case, then disrupting any or all of the MD system would have little or no effect on language comprehension.

These possibilities are difficult to tease apart, and at present we can only rule out the first possibility and some versions of the third possibility. In particular, suppressing the activity of the non-language-dominant hemisphere, including of course the MD regions in that hemisphere, during the intracarotid sodium amobarbital procedure (i.e., the “Wada test”; Wada, 1949), does not appear to greatly affect linguistic abilities (e.g., Rasmussen and Milner, 1977). Of course, it is important to keep in mind that the kinds of language tasks used during the Wada procedure vary substantially across labs and perhaps do not include the most sophisticated tasks currently available for assessing linguistic abilities. Nevertheless, the fact that patients with an anesthetized non-language-dominant (typically, right) hemisphere can understand spoken commands, name pictures, read sentences and repeat phrases, suggests that the core linguistic abilities are preserved. Similarly, removal of the right hemisphere in adulthood impairs many cognitive abilities (e.g., visuo-spatial functions) but leaves linguistic processing largely intact (e.g., Basser, 1962; Searleman, 1977). These results therefore suggest that the MD regions in the non-language-dominant hemisphere are not necessary for linguistic processing. They further constrain the third possibility such that *only the language-dominant-hemisphere MD regions* can be part of the system that is critical for language comprehension. Below I discuss the evidence for why they may or may not be.

## Narrowing down the hypothesis space for the relationship between language processing mechanisms and cognitive control mechanisms

That domain-general cognitive control mechanisms are sometimes engaged during language comprehension is not under debate (see e.g., Novick et al., 2010, for a recent review of the literature). Over the years, abundant evidence has been provided for the connection between working memory and cognitive control resources on the one hand, and language comprehension, on the other hand. This evidence comes from both (a) behavioral studies in healthy and brain-damaged individuals, and (b) brain imaging investigations. For example, in behavioral work, super-additive processing difficulty has been observed in dual-task paradigms that include a language comprehension task and a secondary demanding non-linguistic task (e.g., Wanner and Maratsos, 1978; Waters et al., 1987; Just and Carpenter, 1992; Waters and Caplan, 1996; Gordon et al., 2002; Fedorenko et al., 2006, 2007); associations between language comprehension and executive function abilities have been reported in individual-differences investigations (e.g., Baddeley et al., 1985; King and Just, 1991; Gernsbacher, 1993; Daneman and Merikle, 1996; De Beni and Palladino, 2000; Seigneuric et al., 2000; Burton and Daneman, 2007; Carretti et al., 2009; Novick et al., 2009; Cragg and Nation, 2010; Khanna and Boland, 2010; Gibson and Fedorenko, 2011; McVay and Kane, 2012; Astheimer et al., 2014; cf. Caplan and Waters, 1999); and the depth of linguistic processing has been shown to be affected by top–down reader goals (e.g., Wotschack, 2009). Furthermore, one prominent class of syntactic complexity accounts explains across-construction variability in processing complexity in terms of differential working memory demands (e.g., Wanner and Maratsos, 1978; Gibson, 1998, 2000; Gordon et al., 2002; McElree et al., 2003; Lewis et al., 2006).

Similarly, numerous fMRI studies have reported activations during language comprehension tasks in the domain-general brain regions of the MD system, i.e., in the same brain regions that get modulated by working memory and cognitive control demands (e.g., Duncan and Owen, 2000). A wide range of language phenomena have been shown to produce such activations (often in addition to *also* activating the language regions). These include: non-local syntactic dependencies, especially in older populations (e.g., Peelle et al., 2010; see Kaan and Swaab, 2002; Rogalsky and Hickok, 2011 for a discussion of syntactic complexity manipulations in terms of domain-general factors), ambiguous words or constructions (e.g., Rodd et al., 2005; Novais-Santos et al., 2007; January et al., 2009; McMillan et al., 2013), pronouns whose referents may not be clear from the context (e.g., McMillan et al., 2012), sentences that contain grammatical errors (e.g., Kuperberg et al., 2003; Nieuwland et al., 2012), speech presented under noisy conditions (e.g., Wild et al., 2012), etc.

One possible generalization—based on both behavioral and brain imaging evidence—is that domain-general mechanisms are recruited when difficulties arise in language comprehension, which can of course happen for many reasons. Given that difficulty manipulations across a wide range of cognitive tasks have been shown to produce activity in the regions of the MD system, perhaps the engagement of these circuits during comprehension difficulties in language is not too surprising. Nevertheless, this body of literature is important in that it convincingly establishes that the language interpretation system is not encapsulated (cf. Fodor, 1983) but rather can interact in a flexible way with domain-general working memory and cognitive control mechanisms.

Now, on to the two questions whose answers would help us better understand the precise nature of the relationship between language understanding and domain-general cognitive control mechanisms.

### How frequently do cognitive control mechanisms get engaged when we understand language?

Given that most evidence for the engagement of cognitive control mechanisms in language comprehension comes from cases where language processing is effortful, let us consider how often comprehension difficulties arise in naturalistic linguistic exchanges. For example, how frequently do we encounter ambiguous words whose meaning is not fully constrained by the preceding context? What about non-local dependencies between words? Or cases where we have to rely on a single cue (e.g., word order) to interpret the propositional content of an utterance? Although it is difficult to quantify the proportion of such phenomena in typical linguistic exchanges, corpus analyses suggest that linguistic phenomena that many studies in the field of sentence processing have focused on may not be very common. For example, Piantadosi et al. (2012) demonstrated that ambiguous words are typically used in contexts that strongly favor the relevant meaning. Collins (1996; also Temperley, 2007) has shown that most linguistic dependencies are between adjacent elements (see also Frank et al., 2012). And Roland et al. (2007) showed that object-extracted relative clauses with two full animate noun phrases (e.g., “The senator that the reporter attacked was tall”)—perhaps the most frequently investigated construction in the study of syntactic processing—rarely occur.

Indeed, typical linguistic input abounds with cues to meaning, including lexical information, syntactic information, plausibility/world knowledge information, linguistic and non-linguistic (e.g., visual, social) context, and prosodic/punctuation cues. During the last 30 years, research in the field of sentence processing has established that comprehenders rationally use all the information sources available in the input to derive an interpretation of an input string (e.g., Trueswell and Tanenhaus, 1994; Gibson and Pearlmutter, 1998). It is therefore possible that the focus on controlled manipulations that alter the statistics of the human language has led us to overestimate the importance of domain-general working memory and cognitive control mechanisms in understanding language.

However, comprehension difficulty is of course not categorical. Instead, it varies continuously as we perceive linguistic input and is determined by some combination of (i) how expected the input is from the preceding context, and (ii) how much memory is required for integrating the incoming element into the evolving structure/meaning representation (e.g., Demberg and Keller, 2008; Levy, 2008; Gibson et al., 2014; Levy et al., 2014). The question then becomes: What does it take for the domain-general mechanisms to “kick in” during language understanding? In particular, is the MD system (or some subset of it) always active when we perceive language? Does this system get engaged only when we pay attention to the linguistic input, i.e., when the information in the linguistic signal is somehow relevant to us, or maybe only when some threshold of comprehension difficulty has been reached? Or perhaps we turn to the domain-general mechanisms only in rare cases, as a last resort, when the language system “gives up” on interpretation? Now that we have ample evidence that domain-general mechanisms do sometimes get engaged during language comprehension tasks, we can perhaps focus on understanding the precise conditions under which these mechanisms are recruited, to narrow down these various possibilities.

### Are cognitive control mechanisms necessary for understanding language?

What does the engagement of cognitive control brain regions reflect? Is this engagement functionally important, so that without a properly functioning MD system (or some subset thereof) language interpretation would be severely hampered or impossible? Or is the activation of the MD system simply an “echo” of the effort experienced by the “core” language interpretation system?

To tackle these questions, we need methods that would allow us to examine the effects on language comprehension of inaccessibility of domain-general cognitive control mechanisms. As a result, most evidence from brain imaging investigations does not directly inform these questions. This is also true of much of behavioral evidence, although super-additive processing difficulty observed in dual-task paradigms discussed above does afford some degree of causal interpretation and suggests that taxing domain-general working memory resources can interfere with the processing of (at least syntactically complex) sentences (e.g., Gordon et al., 2002; Fedorenko et al., 2006, 2007). Most direct evidence, however, comes from investigations of individuals with impaired cognitive control abilities. Below I review some of this evidence in light of two alternative positions: cognitive control mechanisms are vs. are not necessary for language comprehension.

#### Evidence for the necessity of cognitive control mechanisms for language comprehension

A few studies have provided evidence of *associations* between difficulties with some aspects of language comprehension and non-linguistic working memory/cognitive control tasks in individuals with brain damage (e.g., Novick et al., 2009; Vuong and Martin, 2011), or with developmental disorders like specific language impairment (e.g., Montgomery, 2003). However, evidence from associations in neuropsychology is notoriously difficult to interpret (e.g., Whitehouse et al., 1978; Caramazza et al., 1982), especially given that domain-general MD regions often lie in close proximity to the regions of the language system (e.g., Fedorenko et al., 2012).

Although I have focused here on cognitive control/working memory, it is important to also consider the role of attention in language comprehension, given that attention is tightly linked to the MD system (e.g., Corbetta and Shulman, 2002; Duncan, 2006). The classic studies of speech perception in the unattended channel (e.g., Cherry, 1953; Broadbent, 1958; Treisman, 1964) showed—across many variations of a similar paradigm—that when presented with two auditory streams and asked to attend to one of the streams listeners are only able to extract minimal information from the unattended stream. Indeed, from mere introspection, we know that when we are not paying attention—whether due to some external stimulus or an internally generated thought—we can “zone out” and miss, for example, a part of a lecture, or a paragraph in a book (see Reichle et al., 2010, for evidence that our eye movement patterns differentiate between the text fragments where we are reading for meaning vs. reading while thinking about something else; also Kaakinen and Hyönä, 2014). These early experimental findings and introspective observations suggest that some minimal amount of attention is necessary to understand language. So to the extent that attention is implemented in the MD system, some minimal MD activity may be required for language comprehension. (Whether this activity is linked to a particular component of the MD system remains to be determined).

#### Evidence against the necessity of cognitive control mechanisms for language comprehension

Perhaps the most compelling evidence comes from the developmental and aging literatures. In particular, the lifetime trajectories of executive and language abilities are different: our cognitive control abilities are slow-developing in childhood, not reaching full maturity until early adulthood (e.g., Kail, 1991a,b,c; Kail and Salthouse, 1994; Harnishfeger and Pope, 1996; Fischer et al., 1997; Munoz et al., 1998; Diamond, 2002; Luciana and Nelson, 2002; De Luca et al., 2003; Luna et al., 2004; Lyons-Warren et al., 2004; Zelazo et al., 2004; Luciana et al., 2005), and yet already at age 5 children can understand impressive amounts of linguistic input (e.g., Kuhl, 2004; Hoff, 2009). In fact, some have argued that the lack of mature cognitive control mechanisms is actually helpful for some aspects of language acquisition (e.g., Newport, 1990; cf. Rohde and Plaut, 1999; Chrysikou et al., 2011). Similarly, although our executive functions decay as we age, our language comprehension abilities remain intact (e.g., Wingfield and Grossman, 2006; Burke and Shafto, 2008), and some abilities (e.g., vocabulary knowledge) keep improving with age (e.g., Field and Gueldner, 2001; Park et al., 2002; Uttl, 2002; Verhaeghen, 2003; Ronnlund et al., 2005). On the extreme end are cases of age-related dementia where some language comprehension abilities remain intact in spite of the steep decline in cognitive control and working memory (Schwartz et al., 1979; cf. Grossman et al., 1996; MacDonald et al., 2001).

Secondly, a number of developmental disorders are characterized by impairments in executive functions with a relative, though almost never complete, sparing of language comprehension abilities, including Turner syndrome (e.g., Money, 1964; Money and Alexander, 1966; Garron, 1970; Murphy, 2009), and select cases of Williams syndrome (e.g., Von Arnim and Engel, 1964; Bellugi et al., 1988, 2000; cf. Karmiloff-Smith, 2006; Mervis and Becerra, 2007) and Down's syndrome (e.g., Evans and Hampson, 1968; Ryan, 1975; Bloom and Lahey, 1978; Rosenberg, 1982; Yamada, 1990; Rondal, 1994, 1995; Anderson et al., 2001; De Luca and Leventer, 2008; cf. Graham and Graham, 1971; Wisniewski et al., 1988). These cases are complemented by rare cases of language savants, individuals with highly impaired general intelligence and allegedly superior linguistic abilities (e.g., Smith and Tsimpli, 1995; cf. Bates, 1997).

Finally, one other line of evidence is worth a brief mention even though it is at present highly controversial (e.g., Laureys et al., 2005). Several reports have suggested that some degree of high-level linguistic processing (e.g., semantic processing) can take place even in patients with severe disorders of consciousness (e.g., Kotchoubey et al., 2002, 2003, 2005; Neumann and Kotchoubey, 2004; Schiff et al., 2005). Given that conscious awareness has been linked to the brain regions of the MD system (e.g., Dehaene and Changeux, 2011), this evidence—if it withstands further evaluation—may be able to provide a strong argument against the need for domain-general cognitive control in at least some aspects of language understanding.

In summary, the evidence for whether cognitive control mechanisms are necessary for us to understand language is at present complex, and more work is clearly needed to answer this question conclusively. The ability to define MD regions functionally at the individual subject level (e.g., Fedorenko et al., 2013) opens to the door to TMS investigations targeting those regions and examining the effects of transient disruption on different aspects of language processing. Furthermore, methods like that pioneered by Woolgar et al. (2010)—where the amount of MD system damage is related to behavioral performance—might prove useful, although such investigations are complicated by the proximity of the MD system to the language system, and thus high probability of damage affecting both systems. In light of the discussion in section How Frequently do Cognitive Control Mechanisms Get Engaged When We Understand Language?, I hope that we—as a field—can expand the scope of the linguistic phenomena we consider when thinking about the role of cognitive control in language. In particular, instead of focusing on language in highly atypical circumstances (e.g., doubly-center-embedded structures or cases where a referent is non-existent), we may want to tackle the more basic question of whether cognitive control is necessary for successful comprehension in typical linguistic exchanges.

## Summary and conclusions

In this paper I have discussed the role of domain-general cognitive control in language comprehension. In recent work we have shown that brain regions that respond robustly to linguistic input are spatially distinct from brain regions that have been linked to working memory and cognitive control (Fedorenko et al., 2011, 2012). These findings suggest that the computations performed by these two sets of brain regions are likely distinct. However, this neural separability of language processing and domain-general cognitive control is compatible with some form of interaction between them, and even with the domain-general circuits being necessary for understanding linguistic input.

Although much evidence suggests that domain-general MD regions are sometimes engaged during language comprehension, it is at present unclear how often this happens, and thus how theoretically significant this engagement is. Moreover, previously reported dissociations between language comprehension abilities and working memory/cognitive control abilities suggest that domain-general mechanisms may not need to function properly for successful language comprehension to occur. However, more evidence is needed to conclusively answer the question of the necessity of cognitive control in language understanding.

The fact that domain-general cognitive control mechanisms may not be necessary for understanding language should not make these mechanisms uninteresting to language researchers, especially given that these mechanisms (a) are important in language production as discussed at the beginning of the paper, and (b) have been implicated in preventing language loss in aging (e.g., Wingfield and Grossman, 2006) as well as in recovery from aphasia (e.g., Sharp et al., 2010). Understanding when and how cognitive control resources are deployed during language comprehension in mature or developing healthy brains may provide important constraints on theories of language acquisition and processing, as well as shed light onto the potential functions of the multiple demand system. For example, even if the MD system is not necessary for language comprehension, it may still turn out to be *useful*, by for example, making language comprehension faster and/or more efficient. A possible analogy is that of a bicycle: although we can get places without one, having one helps us get there faster. According to this view, the MD system is a flexible resource that may get allocated to a wide range of cognitive processes, including those supported by specialized machinery (like face perception or language), and has a beneficial effect on all of those processes. How exactly this facilitation may be implemented is important. For example, do the MD regions simply speed up the processing in the specialized regions by providing extra computational resources of a generic nature (a “workspace”; e.g., Dehaene and Changeux, 2011), or do they provide alternative routes for solving the problem at hand, be it recognizing a face or understanding a sentence?

One intriguing possibility with respect to language—and perhaps other domains—is that the MD system is used for predictive processing. In line with this idea, diminished predictive processing in language has been reported in both children (e.g., Garvey and Berninger, 1981), and aging individuals (e.g., Federmeier et al., 2002, 2010), i.e., groups with underdeveloped and deteriorating cognitive control mechanisms, respectively. There is no question that predictive processing is useful and can speed up the processing of incoming information (e.g., Levy, 2008; Smith and Levy, 2013). However, it is not required: language comprehension can proceed in a bottom-up way, as evidenced by the comprehension abilities of children, elderly individuals, and individuals with otherwise impaired cognitive control mechanisms. This idea—that language regions support a bottom-up language interpretation strategy and MD regions provide a top–down, predictive, strategy for language comprehension—deserves further evaluation.

To conclude, future work should (a) acknowledge that the “core” fronto-temporal language brain regions are spatially and functionally distinct from the domain-general fronto-parietal multiple demand system, and (b) focus on characterizing the circumstances under which domain-general cognitive control mechanisms get engaged during language comprehension, and the precise role of this engagement. Regardless of what the answers to these questions turn out to be, investigations of the relationship between the two systems—including the dynamics of their interaction (see also Fedorenko and Thompson-Schill, 2014)—are likely to inform both, theories of language and of domain-general cognition.

### Conflict of interest statement

The author declares that the research was conducted in the absence of any commercial or financial relationships that could be construed as a potential conflict of interest.

## Footnotes

^1^Stimulus manipulations are not the only way to activate language-responsive brain regions: a number of studies have used task contrasts (e.g., performing a semantic task vs. a control perceptual task on sentences; Ferstl and von Cramon, 2001). Such contrasts presumably force comprehenders to more deeply process the relevant (linguistic) aspects of the signal in the critical vs. in the control, perceptual, conditions.

^2^It is worth noting that referring to these “high-level” language processing brain regions as the “language system” does not imply that no other brain regions are important for language. In fact, we know that sensory regions (both in the auditory cortices—e.g., Belin et al., 2000; Binder et al., 2000; see DeWitt and Rauschecker, 2013, for a recent review)—and in the ventral visual cortex—e.g., Nobre et al., 1994; McCandliss et al., 2003; Baker et al., 2007) play an important role at the early stages of processing auditory/visual language input. Furthermore, regions of the motor and premotor cortex are engaged in articulatory processing (e.g., Bohland and Guenther, 2006). However, I here focus on the higher-level language processing regions and will use the term “language system” to refer to just the latter subset of the language-relevant machinery (see Fedorenko and Thompson-Schill, 2014, for further discussion).

^3^The term “network” is frequently used to refer to sets of brain regions that share functional properties (e.g., Sporns, 2010; Power et al., 2011). However, the available human data do not at present afford strong inferences about anatomical inter-regional connections. So, the term “system” is more accurate, since it is consistent with but does not imply anatomical connectivity.

^4^The notion of a “system” highlights the similarities among a set of brain regions and the differences between these regions and the rest of the brain, but it does not imply a lack of differences among these regions. Different components of the language system may support somewhat different aspects of language processing.

^5^I here have in mind mature adult brains. It is well-known that removal of extensive portions of the brain, including whole hemispheres, in early childhood has few, if any, consequences for cognitive functioning (e.g., Mariotti et al., 1998; Van Lancker-Sidtis, 2004). Similarly, gradual brain deformation due to early-onset disorders like hydrocephalus also often leaves the person largely cognitively intact. These kinds of evidence suggest a high degree of brain plasticity early in development.
