# Supplementary material for: The role of domain-general cognitive control in language comprehension
Source: Front Psychol. 2014 Apr 28;5:335. doi: 10.3389/fpsyg.2014.00335 (PMC4009428; doi:10.3389/fpsyg.2014.00335)
Supplement: Supplementary file 1 [file Presentation1.PDF]

Supplementary Material for: The role of domain-general cognitive control in language comprehension

Evelina Fedorenko

MIT, Brain & Cognitive Sciences Department

43 Vassar Street 46-3037G
Cambridge, MA 02138
evelina9@mit.edu

Supplementary Material

In Fedorenko et al. (2011) we demonstrated that regions of the language system defined by the language localizer task developed in Fedorenko et al. (2010) showed little or no response to several demanding cognitive tasks. However, several objections can be raised with respect to this finding. Here we attempt to parry these objections with several additional analyses.

A. Testing for overlap between language and multiple-demand (MD) regions by examining the responses of the individually defined multiple-demand (MD) regions to sentences and pseudowords.

Motivation for the analysis:

It is possible that some regions of the MD system respond to sentences more than to pseudoword lists – just like the language regions do – but the response is weaker and/or more variable across individuals. This would lead us to miss some language-responsive regions and thus to potentially miss overlap between responses to language and demanding cognitive tasks. We therefore here investigate the possibility of overlap between language and MD brain regions in yet another way, by looking at the response of the MD regions – defined using a task that robustly activates the MD system – to sentences and pseudoword lists. Given that this method is highly sensitive (e.g., Nieto-Castañon & Fedorenko, 2012), we should be able to detect a greater response to sentences than pseudowords if such response is present somewhere in the MD system.

Methods and results:

For this analysis, we used data from 30 participants each of whom performed the language localizer task and a spatial working memory (WM) task from Fedorenko et al. (2011). We used anatomical parcels corresponding to the key MD regions (see e.g., Fedorenko, Duncan & Kanwisher, 2013, for the details of the method) intersected with individual subjects' activation maps for the hard > easy spatial WM contrast to define individual regions of interest (ROIs). (Here and in the analyses reported in Supplementary Material B and C, individual fROIs are defined as top 10% of voxels based on the t-values for the relevant contrast.) We then examined the responses of those fROIs to sentences and pseudoword lists.

As can be seen in Figure A1, although quite a number of MD regions respond above baseline during the processing of sentences, every region responds reliably more strongly to pseudoword lists than sentences (t-values ranging between 1.89 and 6.34; p-values ranging between <.05 and <.0001, FDR-corrected for the number of regions; see also Fedorenko et al., 2013). In other words, MD regions show the opposite response profile with respect to the processing of sentences and pseudoword lists, responding more strongly to linguistically degraded stimuli (pseudoword lists) than to linguistically meaningful and structured stimuli (sentences).

The nature of this effect remains to be determined. One possibility is that the greater response to pseudowords than sentences is due to the fact that the memory-probe task – used in this version of the localizer – is more demanding in the pseudoword-lists condition than in the sentences condition (e.g., Fedorenko et al., 2010). Given that the MD system appears to be sensitive to cognitive effort across diverse tasks (e.g., Duncan & Owen, 2000; Fedorenko et al., 2013), this effect is to be expected. However, even in the passive-reading version of the language localizer we have observed pseudowords > sentences effects in the MD system, albeit somewhat less robustly than in the memory-probe version. This finding is reminiscent of findings in the visual domain where contrasts between non-degraded and degraded visual stimuli activate visual regions in the occipital and temporal cortices, but reverse contrasts (degraded > non-degraded) activate MD regions (e.g., Gorlin et al., 2012). Another possibility is therefore that the response in the MD regions may be a function of how difficult it is to extract meaningful information from the signal (more difficult > greater activation); in contrast, the response in the visual and language regions may be a function of how well incoming stimuli match stored knowledge representations.

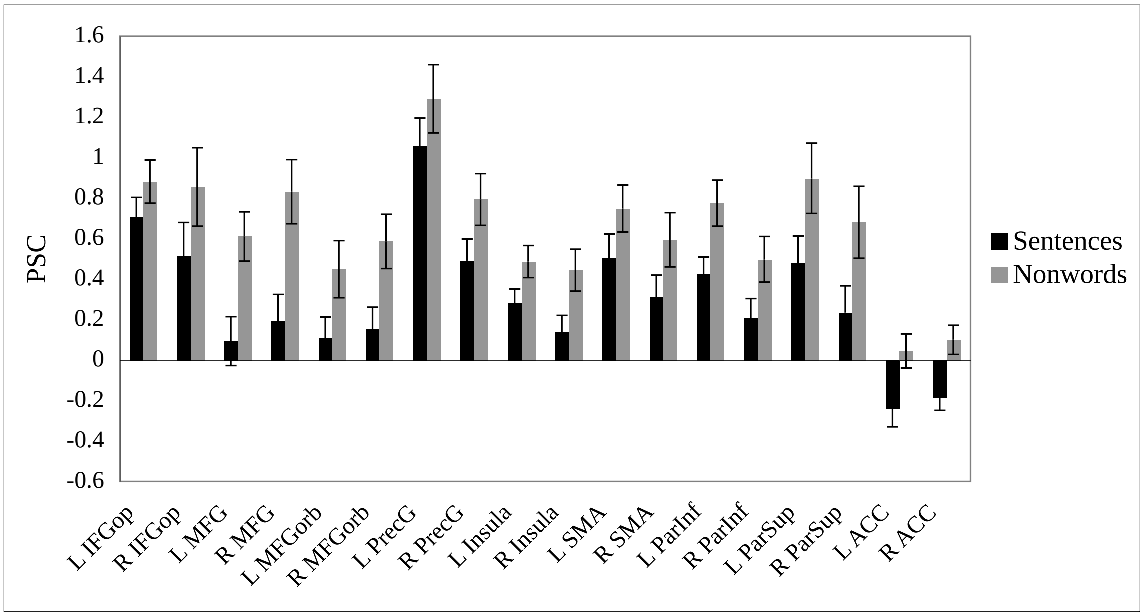

Figure A1. The response of individually-defined MD regions (defined by the hard > easy spatial WM contrast) to sentences and pseudoword lists (n = 30 participants). Every region shows a reliably greater response to pseudoword lists than to sentences, i.e., the opposite of what we find in language regions.

B. Testing for overlap between language and multiple-demand (MD) regions using naturalistic language materials.

Motivation for the analysis:

The sentences in the language localizer task (Fedorenko et al., 2010) may be too simple and may not contain a sufficient number of features that have been shown to cause comprehension difficulty, such as non-local dependencies (e.g., Gibson, 1998; Grodner & Gibson, 2005), lexical and/or structural ambiguity (e.g., Frazier, 1987; MacDonald et al., 1994), or low-frequency words or constructions (Preston, 1935; Forster & Chambers, 1973; Jurafsky, 1996; Levy, 2008). Maybe if more such features were present in the sentences, we would observe greater overlap between language and MD activations, which would manifest as a) a greater response to MD tasks in the language regions, and/or b) a greater response to sentences than pseudoword lists in the MD regions.

Methods and results:

To evaluate this possibility, one strategy would be to construct linguistic materials that abound in features that have been shown to cause comprehension difficulty. However, this approach is not ideal because the resulting materials may not be representative of the kind of input that our language comprehension system receives. Consequently, we decided to use naturalistic materials taken from a corpus of English, which should ensure that the distribution of relevant linguistic features in the experimental materials resembles the one that comprehenders actually encounter in real life.

In particular, we selected ninety-six 12-word-long sentences from the Brown corpus (Kucera & Francis, 1967; these materials are available from EF by request). Each participant (n=8) was run on the language localizer task (Fedorenko et al., 2010) – where the corpus sentences were used instead of the usual lab-constructed sentences – and the spatial WM task discussed above.

First, as in Fedorenko et al. (2011), we defined ROIs functionally in each individual by the *sentences* > *pseudowords lists* contrast, and examined the response of those fROIs to the hard and easy condition of the spatial WM task. In Figure B1 we show the response profiles of these individually-defined fROIs. As in previous work, the responses to sentences and pseudoword lists are estimated in a left-out run not used for ROI definition (e.g., Kriegeskorte et al., 2009). As can be seen from the figure, although the *sentences* > *pseudoword lists* effect is highly robust in left out data (*t*-values ranging between 4.02 and 13.03; *p*-values ranging between <.005 and <.0001, FDR-corrected for the number of regions), none of the regions show any hint of a response to the spatial WM task (*t*s<1.2) for the *hard* > *easy* contrast, or even for *hard* > *fixation* contrast; *t*s<1, except for the LMFG fROI), replicating our finding in Fedorenko et al. (2011).

Next, we defined the MD ROIs in each subject using the contrast between hard and easy spatial WM conditions, and examined the response of those ROIs to sentences and pseudoword lists (see Figure B2). As in the analysis reported in Supplementary Material

A, we found above-baseline responses to sentences in quite a few MD ROIs, but critically, in none of the regions this response was greater than that to the pseudoword lists, and in many regions pseudowords again elicited a greater response than sentences.

These results indicate that the non-overlap between regions that respond to meaningful and structured linguistic stimuli (more than linguistically degraded control stimuli) and those that respond to demanding cognitive tasks (like e.g., the spatial WM task) generalizes to naturally-occurring linguistic materials. So, it is unlikely that we have missed any high-level language-processing regions with our original language localizer contrast.

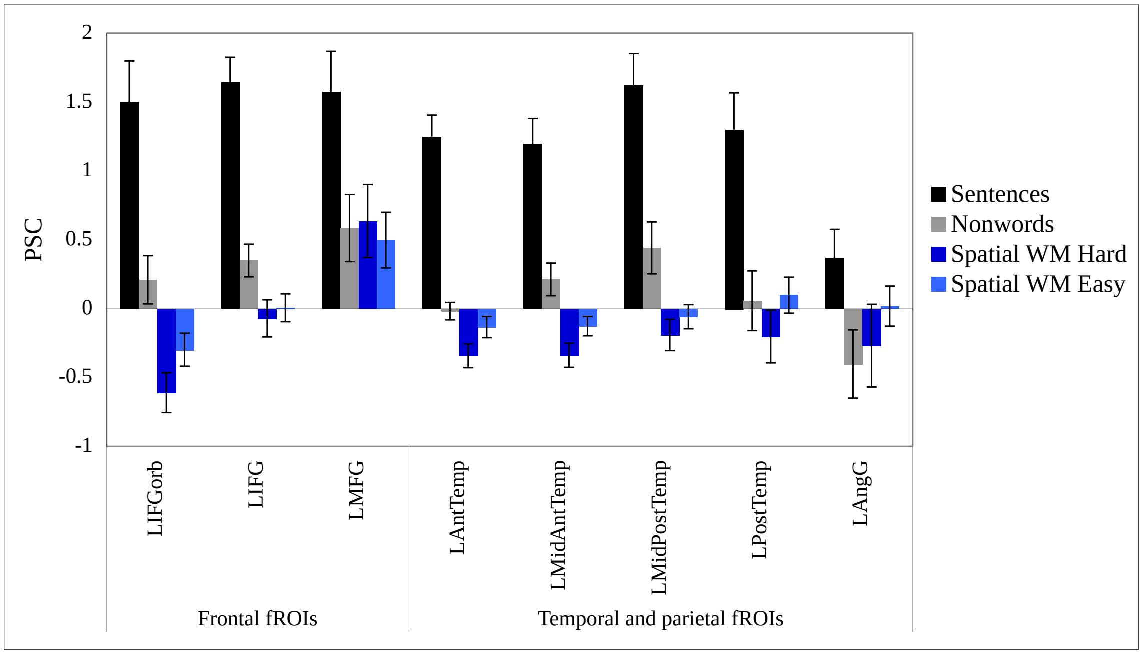

Figure B1. Response of individually defined language-responsive fROIs to sentences and pseudoword lists (estimated using data not used for defining the ROIs) and to the hard and easy conditions of a spatial working memory task (n = 8 participants).

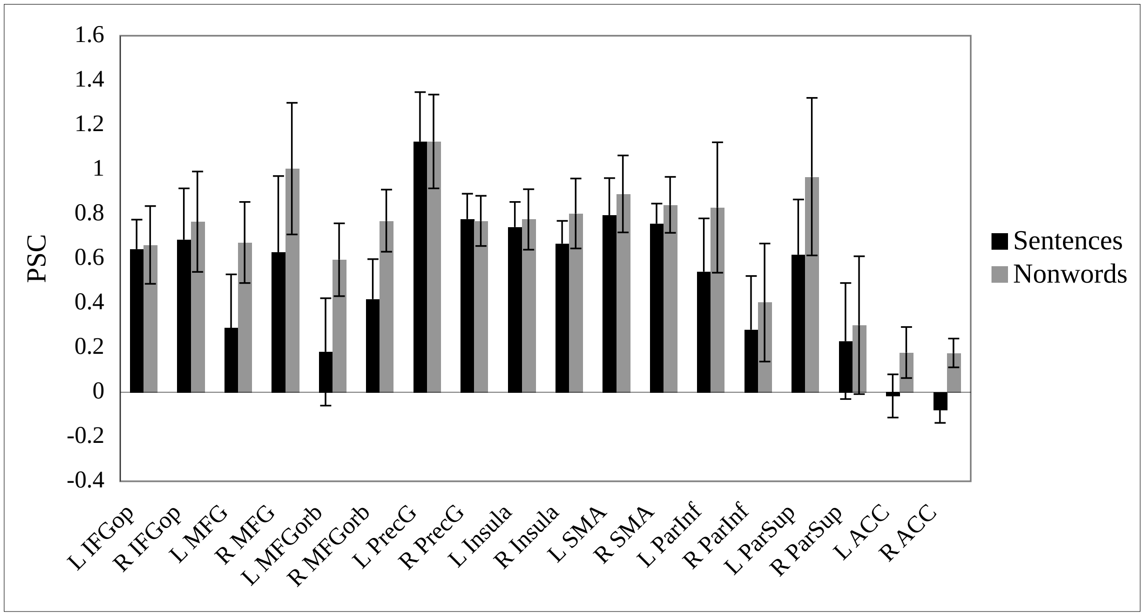

Figure B2. Response of individually defined MD fROIs (defined by the *hard* > *easy spatial WM* contrast) to sentences and pseudoword lists (n = 8 participants).

C. Testing for overlap between language and multiple-demand (MD) regions using the passive-reading version of the language localizer task.

Motivation for the analysis:

In Fedorenko et al. (2011), a memory probe task was used in the language localizer, where after each sentence or sequence of pseudowords participants had to decide whether a probe word / pseudoword appeared in the preceding stimulus. Because this memory probe task is more difficult in the control condition (pseudoword lists) than in the sentences condition (by design; see Fedorenko et al., 2010, for motivation), we may have biased ourselves against finding overlap with demanding tasks because we were excluding regions that respond to general cognitive effort. (Note that this possibility is a priori not very likely given how similar the activation maps are for the localizer with and without a memory probe task (e.g., Fig. 1).)

Methods and results:

To evaluate this possibility, we had four participants perform both the standard version of the language localizer and the version with no memory probe task. The results are shown in Figures C1 (standard language localizer) and C2 (no-task version of the language localizer). Regardless of the version of the localizer that was used for defining the language-responsive ROIs, the results look qualitatively similar to what we have previously observed in Fedorenko et al. (2011), with little or no response to the conditions of the spatial WM task in the language fROIs. (Statistics were not computed because they would not be very meaningful with only 4 participants.)

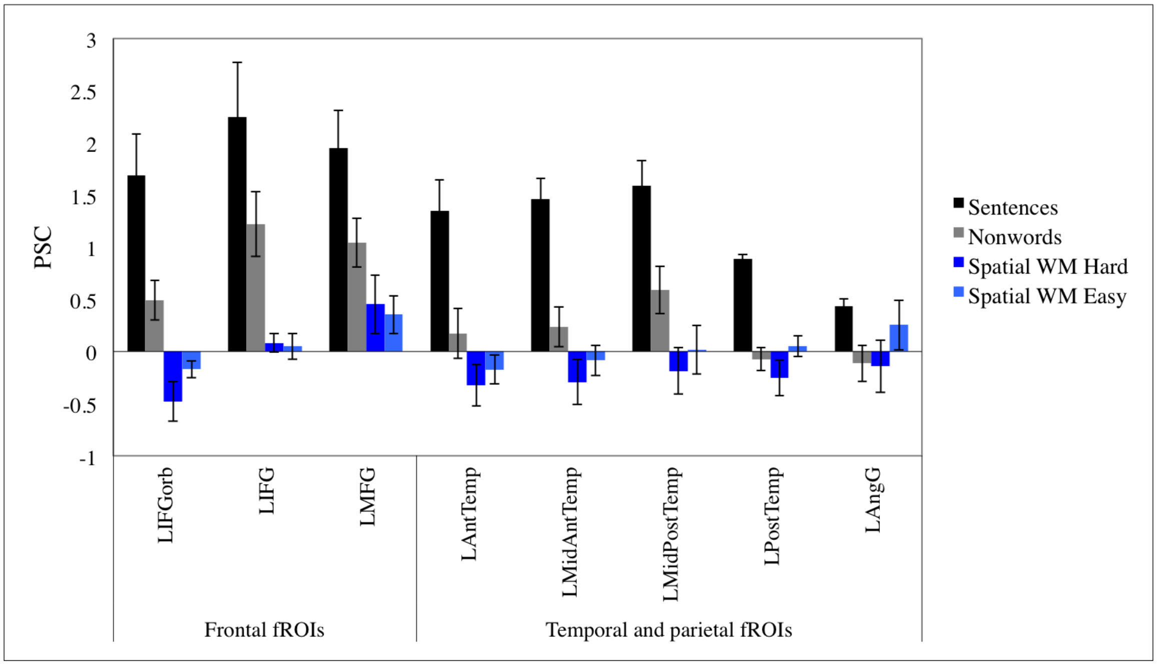

Figure C1. Response of individually defined language-responsive fROIs (using the standard version of the language localizer, with a memory probe task) to sentences and

pseudoword lists (estimated using data not used for defining the ROIs) and to the hard and easy conditions of a spatial working memory task (n = 4 participants).

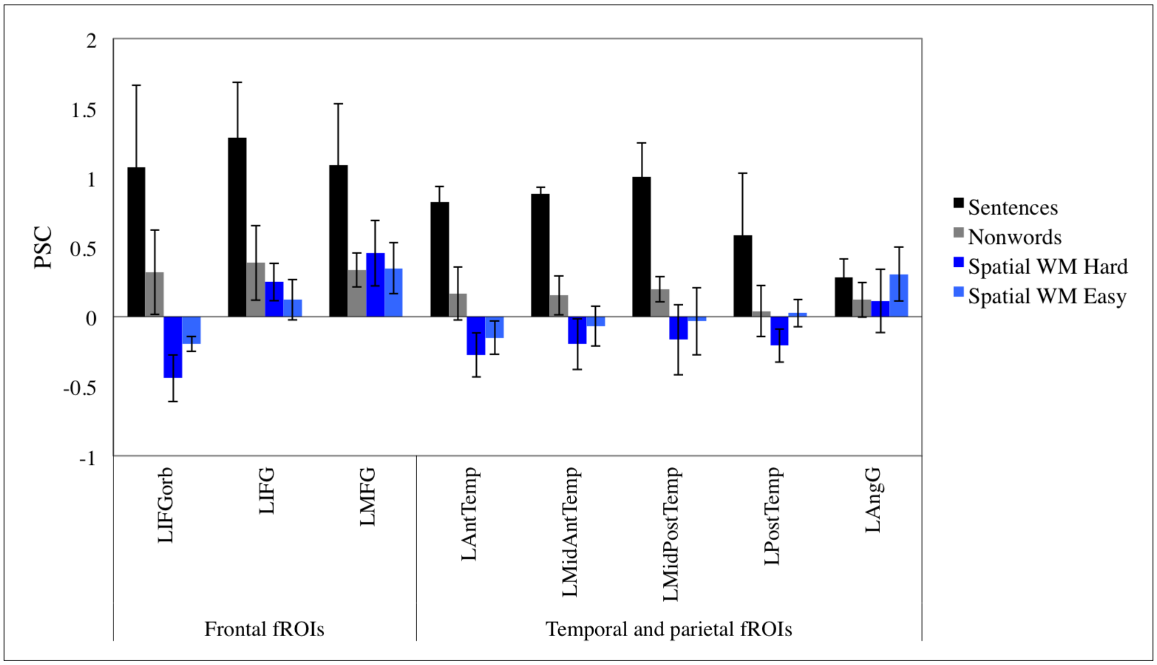

Figure C2. Response of individually defined language-responsive fROIs (using the no-task version of the language localizer; two of the participants performed the visual-presentation version, and two – the auditory-presentation version) to sentences and pseudoword lists (estimated using data not used for defining the ROIs) and to the hard and easy conditions of a spatial working memory task (n = 4 participants).

In summary, language-responsive brain regions are spatially distinct from the domain-general MD system. Unlike the regions of the language system, the MD regions respond at least as much, or more, during the processing of unconnected meaningless elements (pseudowords) as during the processing of sentences, including naturally occurring ones.
